# Few-Shot Rolling Bearing Fault Diagnosis with Metric-Based Meta Learning

**DOI:** 10.3390/s20226437

**Published:** 2020-11-11

**Authors:** Sihan Wang, Dazhi Wang, Deshan Kong, Jiaxing Wang, Wenhui Li, Shuai Zhou

**Affiliations:** School of Information Science and Engineering, Northeastern University, Shenyang 110819, China; 2010257@stu.neu.edu.cn (S.W.); 1910674@stu.neu.edu.cn (D.K.); 1800797@stu.neu.edu.cn (J.W.); 1910244@stu.neu.edu.cn (W.L.); 1610234@stu.neu.edu.cn (S.Z.)

**Keywords:** deep learning, few-shot learning, meta learning, convolutional neural network, bearing fault diagnosis

## Abstract

Fault diagnosis methods based on deep learning and big data have achieved good results on rotating machinery. However, the conventional deep learning method of bearing fault diagnosis is mostly based on laboratory artificial simulation data, and there is an error with actual fault data, which will reduce the generalization performance of the deep learning method. In addition, labeled data are very precious in real industrial environment. Due to expensive equipment and personnel safety issues, it is difficult to obtain a large amount of high-quality fault labeling data. Therefore, in this paper, we propose a metric-based meta-learning method named Reinforce Relation Network (RRN) for diagnosing bearing faults with few-shot samples. In the proposed method, a 1D convolution neural network is used to extract fault features, and a metric learner is used to predict the similarity between samples under different transfer conditions. Label smoothing and the Adabound algorithm are utilized to further improve the performance of network classification. The performance of the proposed method is verified on a dataset which contains artificial damage and natural damage data. The comparison studies with other methods demonstrate the superiority of the proposed method in the few-shot scenario.

## 1. Introduction

Bearings, as rotating machinery, play a very important role in motors and engines. Bearing failure will cause mechanical damage and even threaten the safety of users. Therefore, accurate prediction and diagnosis of various bearing failures in real industrial scenarios is of great significance. In the past few years, a large number of traditional signal processing and machine learning methods have been applied to bearing fault detection, including wavelet transform (WT), Fourier transform, empirical mode decomposition (EMD) [1,2], principal component analysis (PCA) [3], SVM [4], k-nearest neighbor [5], and random forest [6]. Ren [7] proposed a 3-D waterfall spectrum in combination with reassigned wavelet scalogram method to solve non-linear and non-stationary vibration signal, while Yan [8] proposed a novel multiscale morphology analysis method, which can preserve signal details and has a good performance in detecting the defects in bearing. However, the conventional fault diagnosis method has some disadvantages:

(1) Based on signal processing and conventional machine learning methods, a large number of manual feature extraction operations are required, which cannot adapt well to the complex dynamic system of bearing vibration signals;
(2) Conventional machine learning methods cannot learn the nonlinear relationships in the system;
(3) Artificial feature extractor and expert systems cannot extract fault features well against changing scenario data, and sufficient expert knowledge of signal processing is usually required, which is not convenient for industrial applications.

To address these problems, deep learning methods are widely used in intelligent fault diagnosis, from the model perspective, including Convolution Neural Network (CNN) [9], Recurrent Neural networks (RNN) [10], Deep Boltzmann Machine (DBM) [11] and autoencoders (AE) [12]. This is because the convolutional neural unit works like a filter and is specifically designed for highly nonlinear and complex signals, and the CNN has been widely used in the fault diagnosis of bearings [13,14,15], gears [16,17,18] and other rolling elements, Li [19] proposed a domain adaptation method for machinery fault diagnostics across sensors at different places, Zhang [20] proposed a residual learning algorithm to solve gradient vanish and gradient explode problem which improved network training. However, conventional deep learning methods need a large amount of high-quality labeled data to obtain a good performance. However, it is difficult to collect enough labeled data in the real industrial environment, especially lack of fault data. In addition, most of the deep learning models are trained and tested in the laboratory’s artificially simulated environment, and there is still a gap with real industrial applications.

**Figure 1:**
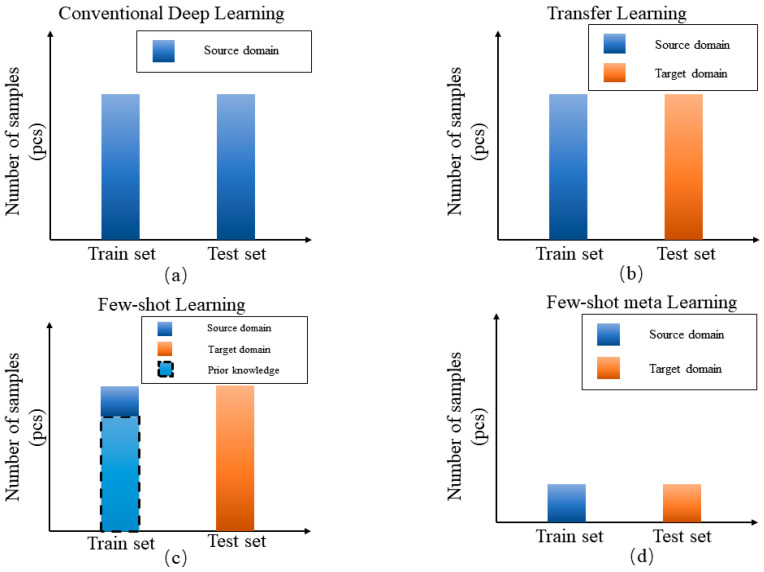
Different scenarios for deep learning bearing fault diagnosis.

The scenarios of traditional deep learning, transfer learning, traditional few-shot learning and few-shot meta learning are shown in Figure 1, different colors represent different domains, prior knowledge in few-shot learning are denoted with the dotted box. Conventional deep learning assumes that the train set and test set are under the same domain and consist of sufficient labeled samples. Transfer learning is where the train and test set are under related domains. For conventional few-shot learning, it can train using related large-source domain samples as prior knowledge and some task-specific samples for the N way K shot problem, then test in a few-shot target domain. In few-shot meta learning, only a few samples are given from the training set, for problems such as few-shot scenario and conditions transfer can be effectively solved by few-shot meta learning technology. Based on the above reasons, this paper proposes a Metric-based few-shot meta-learning technology to be applied to bearing fault diagnosis and verified on artificial-natural bearing datasets. The results show that the proposed method is better than conventional methods in few-shot scenarios. The contribution points of this article are summarized as follows.

1. Propose a metric-based few-shot meta learning method for bearing fault diagnosis;
2. Label smoothing is adopted to alleviate over-fitting and improve generalization in few-shot learning;
3. Adabound is first introduced in fault diagnosis, which can converge faster and obtain higher accuracy.

The rest of this article is organized as follows. Section 2 introduces few-shot learning and meta learning. Section 3 elaborates on the proposed method. Section 4 introduces label smoothing and Adabound. In Section 5, the proposed method is evaluated by the artificial-natural bearing datasets. Finally, Section 6 concludes this article.

## 2. Background

### 2.1. Few-Shot Learning

**Figure 2:**
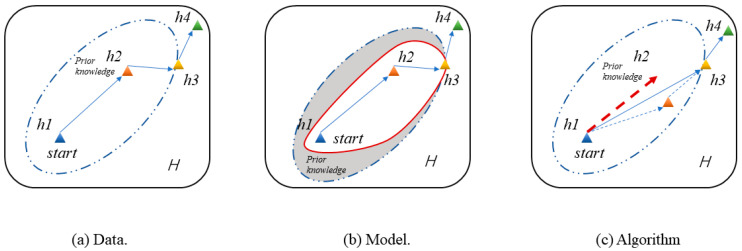
Different categories of few-shot learning.

Machine learning has been highly successful in data-intensive applications, but is often hampered when the dataset is small [21]. However, humans can learn to recognize a new object or master a new concept with only one- or few-shot instances; the essential reason for this is that humans can use prior knowledge to learn. Inspired by this, few-shot learning is proposed, based on prior knowledge, to learn data features in few-shot scenarios, to solve the prediction and classification problems in the case of missing data [22]. Few-shot learning is a type of machine learning problem, where experience contains only a limited number of examples with supervised information for the target task. Few-shot learning can be divided into three categories based on current research progress: data, models and algorithms [23]. As shown in Figure 2a, data-based method can use prior knowledge to augment the raw data from *h1* to *h2*; with sufficient data, conventional deep learning methods can be used. For the model-based method, in Figure 2b, prior knowledge is used to constrain the complexity of *H*, where *H* is the hypothesis space determined by the model, and *h4* is the optimal hypothesis from data to label. In Figure 2c, an algorithm-based method is the optimization strategy which uses prior knowledge to search through *H* in order to find the best hypothesis *h3* in *H*.

Data augmentation technology has been used extensively in tasks such as computer vision and natural language processing in the past. In the field of bearing fault diagnosis, Zhang [24] performed data augmentation by manually copying and intercepting the original signal, Li [25] used Generative Adversarial Networks (GAN) to solve the problem of category imbalance, Gao [26] used a combination of finite element (FEM) and GAN, not only to supplement the number of missing labeled data, but also to supplement the missing attributes, and Cubuk [27] described a simple procedure called AutoAugment, which automatically learns the augmentation policy for deep network training. The core idea of the above method is based on the existing labeled data, that is, prior knowledge, to create similar labeled data or copy directly according to the extracted features, so as to train the neural network on a large amount of labeled data to obtain a good performance. However, the augmentation rules can be specific to the dataset, making them hard to apply to other datasets. Therefore, manual data augmentation cannot solve the FSL problem completely [28]. Moreover, the GAN-based data augmentation method has disadvantages, as the training and generation results of GAN are not robust enough.

The most common model-based, few-shot learning technology is embedding learning [29]: the training set and testing set are denoted D_train_ and D_test_, the embedding function F projects the training sample data X_train_ ∈ D_train_ to a low-dimensional space Z, the embedding function g projects the testing samples X_test_ ∈ D_test_ to Z, then a similarity equation S is used to predict the embedding similarity between classes. Zhang [30] used a Siamese network for bearing fault diagnosis, Vinyal [11] proposed a matching network as a semi-supervised method to assign unlabeled samples to augment D_train_ via soft-assignment during learning, Sung [31] used a relational network to embed samples into the status space at the same time, and used a convolutional neural network to automatically find similarities between different image categories, and Snell [32] proposed a prototypical network, Instead of comparing *f* (x_test_) with each *f*(xi) where xi ∈ D_train_, the prototypical network only compares *f* (x_test_) with the class prototypes in D_train_. For class n, the prototype is calculated by the formula

(1) $$C_{n}=\frac{1}{K}\sum_{i=1}^{K}f(x_{i})$$

where X*_i_* is one the *K* examples of the nth class in D_train_, and f is the embedding function.

The algorithm-based method uses strategies to find better initialization parameters more quickly or refine existing parameters. Hinton [33] proposed a fine-tuning based method; this strategy takes the $\theta_{0}$ of a pre-trained model learned from related tasks as a good initialization, and adapts it to θ by D_train_. The assumption is that $\theta_{0}$ captures some general structures of the large-scale data. Therefore, it can be adapted to D_test_ with a few iterations. Zhang [34] proposed a few-shot learning approach named model-agnostic meta-learning (MAML), which improve the efficiency of the model. Nichol [35] proposed a new algorithm called reptile, which can obtain a better initialization parameter than MAML and pre-training.

All the above few-shot learning methods have been widely used in the field of CV and NLP, However, few-shot learning in the field of rotating machinery fault diagnosis is still very scarce. In order to further develop the few-shot fault diagnosis of bearings, it is necessary to explore the applicability of the few-shot learning method in bearing fault diagnosis and the advantages and disadvantages compared with conventional methods.

### 2.2. Few-Shot Meta Learning

Meta learning is one of the most promising and trending research areas in the field of artificial intelligence right now. It produces a versatile AI model that can learn to perform various tasks without having to train them from scratch, and this method is widely used in the field of few-shot learning, also known as few-shot meta learning. We can categorize meta learning into three categories: metric-based meta learning, initialization-based meta learning and optimization-based meta learning [36].

Metric-based meta learning will learn the similarity between different classes. It uses a neural network to extract the features from a dataset and finds the similarity by computing the distance between different features of these classes, such as Siamese networks, prototypical networks and relation networks. Initialization-based meta learning will initialize the weights with optimal values or close to optimal values; by using this method we can attain the convergence faster, such as MAML, Meta-SGD [37] and reptile. Optimization-based meta learning will have two networks: one base network that actually tries to learn and a meta network that optimizes the base network. Mainstream research methods of optimization-based meta learning include the LSTM Meta-Learner [38].

## 3. Model Framework

### 3.1. Data Preprocessing

During the raw data preprocessing, time shift, the frequency resolution of the vibration signal needs to be considered. Since the neural network cannot satisfy the time-shift invariance of the vibration signal, it is necessary to convert the original vibration signal to the frequency domain through Fast Fourier Transform (FFT) to solve this problem. For the frequency resolution (FR), it is necessary to intercept a signal of sufficient length to ensure the FR, but, at the same time, the model capacity and computational overhead must be considered, so, after trade-off, the input length of models is uniformly set as 1024. For the random noise carried by the data, in the preprocessing module, methods such as Gaussian filter [39] are used, and methods such as increasing the first layer convolution kernel [24], and threshold denoising algorithm [40] are used in the model module. This article mainly uses [24] for anti-noise processing.

### 3.2. Network Structure

Since the transfer learning with pre-training and fine-tuning is very similar to the few-shot learning, this paper will compare two methods; the same 1D convolutional neural network structure, pooling layer, batch standardization and activation function will be used in both networks. In the first layer, a large convolution kernel will be adopted to capture more shallow features while reducing the influence of high-frequency noise [41]. Both networks will use the same feature extractor; the full connection layer and Softmax is used as the classifier in transfer learning; the convolutional neural network is applied to the metric learner of the few-shot learning method.

### 3.3. Methods

**Figure 3:**
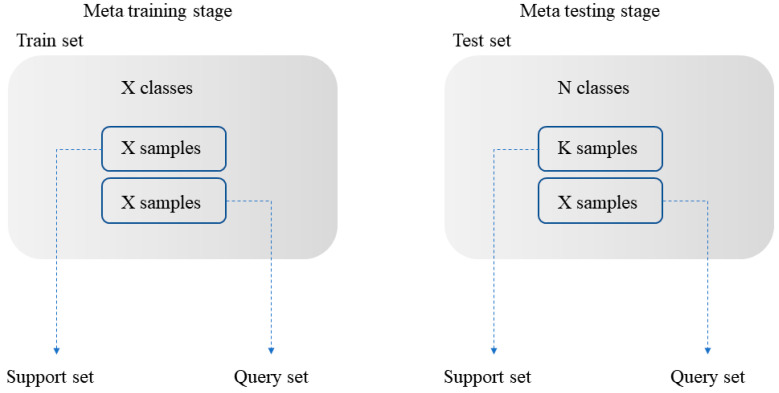
N way K shot classification problem.

The transfer learning method based on pre-training and fine-tuning trains the data with a large number of labeled data, and then fine-tunes the network on a specific category; the weight of feature extractor is fixed and only the classifier is trained. Based on the few-shot meta learning, the data are divided into a meta training stage, which becomes the source domain, and the meta testing stage also becomes the target domain, among which both are divided into a support set and query set. The support set in the two domains is used to calculate the prototype of data features, and the query set in the two domains is used to train and improve the model performance. As shown in Figure 3, a common N way K shot problem is that N categories are selected on the support set of the meta testing stage, and K samples are selected for each category, without limiting the capacity of the other set.

#### 3.3.1. Transfer Learning

Pre-training refers to training on the source domain to obtain a feature extractor, and fine-tuning refers to fixing the feature extractor on the target domain and training the classifier. The feature encoder is learned in the source domain by Adam optimizer with the learning rate of 0.001, the training epoch is 100, and a new classifier is trained in the target domain with the momentum-accelerated stochastic gradient descent with the learning rate of 0.01, the N way K shot task is divided into a minibatch, and trained with 50 epochs, and the average accuracy of the last ten times is taken, and the training will be repeated 20 times to offset the randomness of sampling.

#### 3.3.2. Few-Shot Meta-Learning

The few-shot meta-learning in this paper will adopt a metric-based meta-learning method: Reinforce Relational Network (RRN). In solving the few-shot problem, the relational network has achieved state-of-the-art results in the metric-based meta-learning model. The feature extractor of the RRN is the same as the transfer learning method: the metric learner consists of two convolutional layers and two fully connected layers, samples of the support set and query set are processed by feature extractor and embedded into the same feature space, the similarity of the two sets was computed by metric learner and the similarity value was predicted.

**Figure 4:**
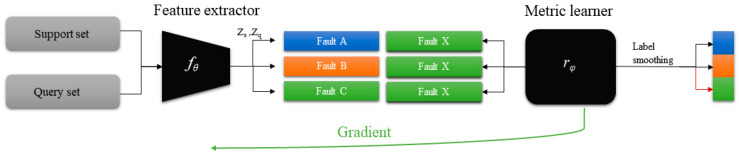
Learning procedure of few-shot meta learning with the episodic training strategy.

As shown in the Figure 4, meta learning usually uses a strategy called episodic training [42]. The training procedure, support set and query set are extracted by CNN module and imported into the classifier, where the objective is learning the projection function $r_{\varphi }(f_{\theta }(x))$ between data and labels, which can be formalized as follows

(2) $$r^{*},\theta^{*}=\underset{\theta }{\arg\min}(\sum_{}^{}Loss(f_{\theta }(x),c));x\in D_{s}^{source}$$

where $f_{\theta }$ is feature extractor, Z_s_ and Z_q_ are embedding vectors of the support set and query set.

The average of the 100 episodes is finally extracted as the final accuracy, and the training will be performed 20 times to offset the randomness of the sampling.

## 4. Label Smoothing and Adabound

### 4.1. Label Smoothing

Due to the overfitting problem of few-shot learning, the generalization ability of the model is reduced. In addition, the original data will be mixed with an abnormal signal, and the one-hot encoding will lead to the model learning abnormal characteristics, and reduce its generalization ability. Therefore, this article uses label smoothing to alleviate the above problems [43]. The core of label smoothing is to modify the ground-truth label

(3) y_ls=(1−α)*y_hot+α/K

where K is the number of label classes, and α is a hyperparameter that determines the amount of label smoothing. If α = 0, we obtain the original one-hot encoded *y_hot*. If α = 1, we obtain the uniform distribution. The purpose of label smoothing is to soften one-hot encoding. The content of one-hot encoding only includes 0 and 1, which will cause the model to trust the raw data too much. If the raw data contain abnormal data, it will seriously affect the model effect and reduce the robustness. As can be seen from the above equation, when *y_hot* equals 0, *y_ls* equal to α/K, which is bigger than 0. When *y_hot* equals 1, *y_ls* equals 1 − α + (α/K), which is smaller than 1. By learning the soften value rather than the ground-truth label, the model can alleviate the problem of over-fitting.

### 4.2. Adabound

Stochastic gradient descent (SGD) and Adam have been proposed to achieve a rapid training process with an element-wise scaling term on learning rates. However, due to the unstable and extreme learning rates, there is a chance of causing performance degradation. By dynamically tailoring the learning rate, Adabound [44] can alleviate this problem. The core of the Adabound algorithm is to limit the rate of adaptive learning

(4) $$Clip(\alpha /\sqrt{V_{t}},\eta_{l},\eta_{u})$$

where α is the initial step size, $\alpha /\sqrt{V_{t}}$ is the learning rate of the algorithm, and $\eta_{l}$ and $\eta_{u}$ are the upper and lower bounds of the output. Adabound employ dynamic bounds on learning rates in these adaptive methods, where the lower and upper bound are initialized as zero and infinity, respectively, and they both smoothly converge to a constant final step size. This helps the model to converge faster and obtain higher accuracy.

## 5. Case Study

**Figure 5:**
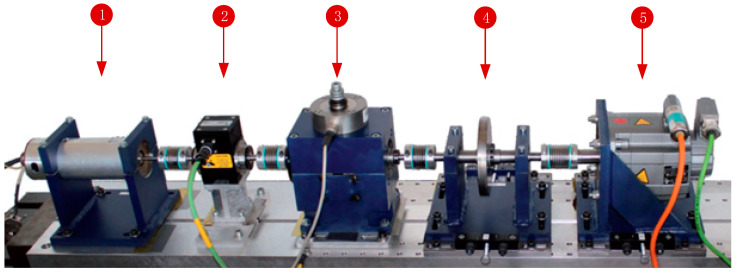
Mechanical setup of the test rig: (1) test motor; (2) measuring shaft; (3) bearing module; (4) flywheel; (5) load motor.

Paderborn University (PU) bearing datasets were provided by Lessmeier, C et al. [45] for condition monitoring and bearing fault diagnosis based on vibration and motor current signal. A total of 32 bearings of type 6203 were included, including six undamaged (healthy) bearings, 12 artificially damaged bearings, and 14 real damages caused by accelerated lifetime tests. Each of the 32 fault categories were tested on four different working conditions with different combinations of rotational speed, load torque, and radial force. All bearings were installed in the modular test rig for a unified test; the modular setup used to collect the PU bearing dataset is shown in Figure 5.

**Figure 6:**
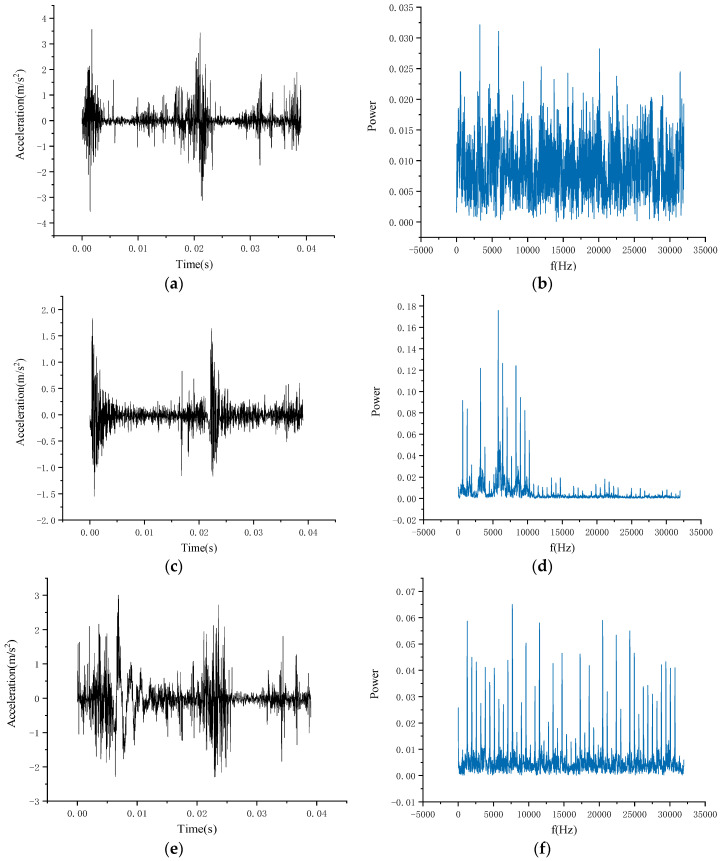
Visualization of signal in time domain and frequency domain. (**a**,**c**,**e**) are time domain images of signal, (**b**,**d**,**f**) are the frequency domain images of signal.

Since most of the current deep-learning-based fault diagnosis methods rely on simulated data in the laboratory, there is still a gap with the real situation. To solve this problem, this paper will conduct training on simulated data and test on real data, which can not only alleviate the problem of a small amount of real data, but also improve the accuracy of model transfer to real situations. The PU bearing dataset has artificial simulation and real bearing fault signal, which can help us complete this experiment. The visualization of signal in time domain and frequency domain is shown in Figure 6. Figure 6a,b shows healthy bearings, Figure 6c,d shows bearings from the artificial damage set, and Figure 6e,f from the natural damage dataset.

**Figure 7:**
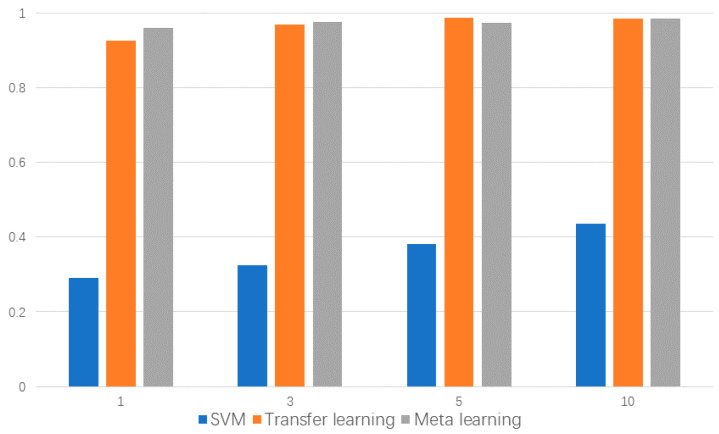
Accuracy comparison on PU bearing dataset.

To perform artificial-to-natural few-shot meta learning, we selected 13 representative classes from the total 32 classes, with having a 1 healthy bearing, 8 artificial damaged bearings, and 4 real bearing failures resulting from accelerated lifetime testing. The test rig ran at n = 900 rpm with a load torque of M = 0.7 Nm and a radial force on the bearing of F = 1000 N; sampling frequency was 64 KHz and the vibration signal was perpendicular to the axis. The details of these 13 bearings are shown in Table 1. We conducted few-shot meta learning training on eight artificial damage categories, and tested under four real damages and one healthy state. Each category was tested with 1, 3, 5, 10 shots, and the accuracies are shown in Figure 7. Although Zhang et al. [30] proposed a few-shot learning strategy for bearing fault diagnosis, since the training and testing sets are of the same class, its essence is still a variety of transfer learning methods based on prior knowledge and fine-tuning.

It can be seen that the few-shot meta learning method achieved the best accuracy in the 1-shot and 3-shot situation. In the 5-shot situation, the transfer learning method achieved the best accuracy. Meanwhile, in the 10-shot situation, the performance of the two methods is almost the same. The worst accuracy is also given with transfer learning in the 1-shot situation; the above results indicate that few-shot meta learning can effectively learn sample features in the case of extreme lack of samples, and the knowledge obtained from the artificial simulation fault data can be effectively transferred into natural fault data to improve the classification accuracy. Moreover, a statistical method called support vector machines (SVM) was used for comparison. For each training set, SVM used the whole training set to fit the model and achieved the worst results in all scenarios; this may be due to the small amount of data, meaning that the SVM could not learn the distribution of samples, and was unable to find the best hyperplane with the largest geometric margin.

**Figure 8:**
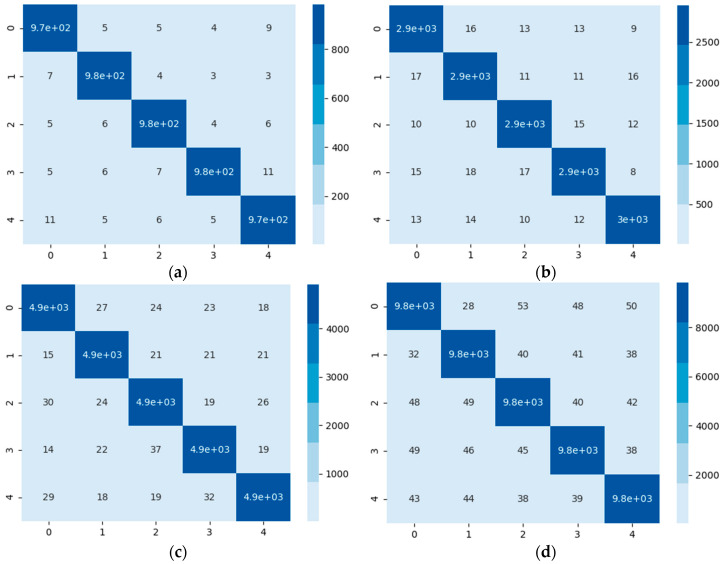
The confusion matrices in different tasks using the proposed method. (**a**–**d**), corresponding to 1-shot, 3-shot, 5-shot and 10-shot tasks.

Furthermore, Figure 8 shows the confusion matrices (a), (b), (c) and (d), corresponding to 1-shot, 3-shot, 5-shot and 10-shot tasks, where the prediction results and the ground truths are presented. Each episode, was repeated 1000 times to offset the randomness of sampling, and the average accuracy of 1000 times was taken.

**Figure 9:**
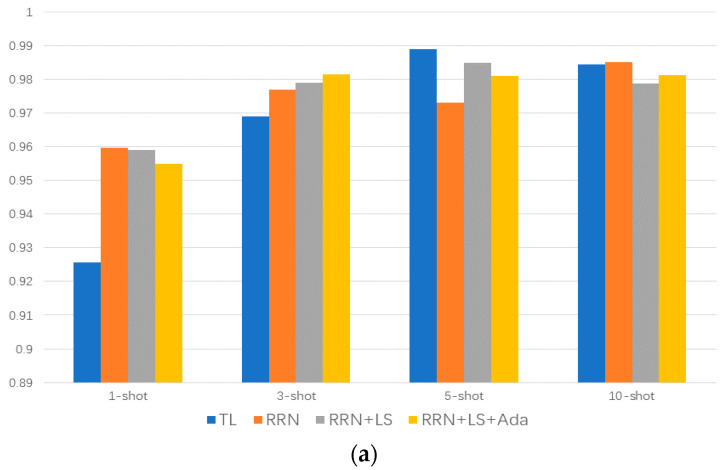

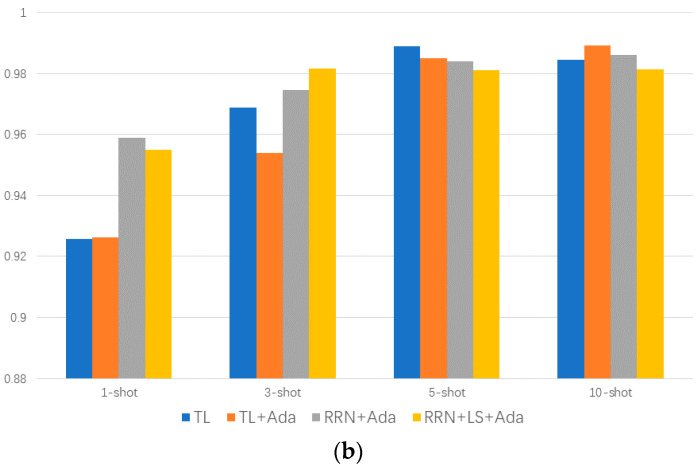
Effect of label smoothing and Adabound on model accuracy. (**a**) represents the comparison of the result accuracy of TL, RRN, RRN with LS, and RRN with LS and Ada, (**b**) represents the comparison of the result accuracy of TL, TL with Ada, RRN with Ada, and RRN with LS and Ada.

Figure 9a shows the differences between RRN and transfer learning (TL) methods under label smoothing (LS) regularization and Adabound (Ada) optimizer improvements. It is seen that the predicted accuracy by the proposed method RRN is better than the result of the transfer learning in the 1-shot and 3-shot situation. The proposed method with label smoothing obtains the mean accuracy of 97.54%, the transfer learning method obtains the mean accuracy of 96.70%, and, compared with the mean accuracy of 97.38% obtained by the proposed method without label smoothing, this indicates that the label smoothing can improve the predicted accuracy effectively.

Moreover, the transfer learning method and RRN with Adabound optimizer is investigated, and the accuracies of the above methods are presented in Figure 9b. It is seen that Adabound is effective only for RRN and is inferior even to the baseline accuracy when added to the transfer learning method. The mean accuracies of the transfer learning method with Adabound, RNN with Adabound, RNN with label smoothing and Adabound are 96.36%, 97.59% and 97.47%, respectively.

**Figure 10:**
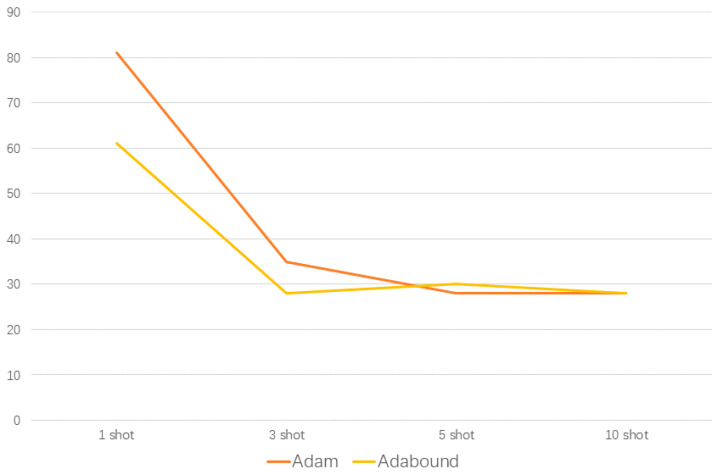
The episodes required to achieve 96% accuracy.

Adabound can not only improve the accuracy of the model, but can also improve the convergence speed of the model. The average number of episodes used to stabilize the accuracy above 96% by using the Adam optimizer and Adabound optimizer is shown in Figure 10. To offset the deviation caused by random sampling, the above results are the average results after 1000 runs for each episode. In the 1-shot and 3-shot scenarios, the average episode required by the Adabound optimizer is 61 and 28, while for Adam it is 81 and 35, so the convergence speed of Adabound is faster. In the case of 5-shots or more, the convergence speed of the Adam and Adabound optimizer is gradually consistent. The metric-based, few-shot meta-learning was implemented by the Pytorch framework under Python 3.5. Training and network testing were performed on a personal computer with Windows 10 operating system, an Intel Core i7-9770F CPU, and a GTX 1660Ti GPU. For each episode, 10.4 s of average training time is required.

Combining the above analyses, RNN with Adabound can dominate in an extremely few-shot situation, and the conventional transfer learning method can dominate as the shot number increases. Furthermore, in this paper, eight fault categories of artificial damage are used for training, and five categories of natural damage are used for testing and verification. For the eight categories in the training set, natural damage is a new fault mode. Therefore, new fault classes can be recognized. However, as the randomness of sampling and the robustness of neural network cannot be proved, it can be seen from the above results that the robustness of the proposed method can be improved. In addition, the explanation for the black box of the neural network also needs further study in the future.

## 6. Conclusions

In this paper, a few-shot meta-learning method for bearing fault diagnosis is studied. The raw signal is converted into a frequency signal by FFT, so the time shift in the vibration signal need not be considered. In situations of transfer from artificial simulation to natural damage, the modules of feature exactor and metric learner are used to extract features and compute the similarity between learning features, respectively, which consequently automatically extracts features and enables classification. Metric-based meta learning methods, regularization methods, and the Adabound algorithm have been proposed and tested on real data. For the sake of fairness, all networks are unified with a 1D convolutional neural network.

- A metric-based, few-shot, meta-learning framework is designed for bearing fault diagnosis, which is more suitable for a few-shot transfer scenario from the experimental situation to the actual working situation;
- Comparison analysis among the designed few-shot meta-learning method and fine-tuning-based transfer-learning method is performed, showing that the proposed method has a better performance in the case of extreme data absence. The proposed method is 5% more accurate than the conventional transfer learning method and 65% higher than the conventional statistical method in extremely few-shot scenarios;
- The label smoothing regularization method and Adabound optimizer can inhibit the overfitting in the learning process of small sample elements. The Adabound optimizer can help the model learn the data feature more quickly, and reduce mode training by up to 20 episodes.

In future work, we will further improve the stability and interpretability of the model, and reduce the number of hyper-parameters. In recent years, the few-shot meta learning method has been increasingly applied in fault diagnosis, which is a potential method for bearing fault diagnosis with few samples and condition transfers. The effectiveness of the proposed framework makes it a promising method for fault diagnosis.

## Figures and Tables

**Table 1:** Details of Paderborn University (PU) bearing dataset.

Bearing Name	Fault Location	Damage	Severity
K001	Healthy	Healthy	Healthy
KA01	OR	EDM	1
KA03	OR	EE	2
KA05	OR	EE	1
KA07	OR	Drilling	1
KA08	OR	Drilling	2
KI01	IR	EDM	1
KI03	IR	EE	1
KI07	IR	EE	2
KA04	OR	pitting	1
KB23	OR + IR	pitting	2
KB27	OR + IR	plastic deform	1
KI04	IR	pitting	1
OR: outer ring	IR: inner ring		
EMD: Electrical discharge machining			
EE: Electric engraver			
